# Association of Scoliosis and Severe Knee Osteoarthritis: A Case-Control Study

**DOI:** 10.3390/jcm13237369

**Published:** 2024-12-03

**Authors:** Conmin Chen, Kuang-Ting Tsai, Kuang-Ting Yeh, Shuo-Suei Hung

**Affiliations:** 1 Department of Medical Education, Taipei Tzu Chi Hospital, Buddhist Tzu Chi Medical Foundation, New Taipei City 231, Taiwan; tch38109@tzuchi.com.tw; 2 Department of Medical Education, National Cheng Kung University Hospital, College of Medicine, National Cheng Kung University, Tainan 701, Taiwan; n172017@hosp.ncku.edu.tw; 3 Department of Orthopedics, Hualien Tzu Chi Hospital, Buddhist Tzu Chi Medical Foundation, Hualien 970, Taiwan; micrograft@tzuchi.com.tw; 4 School of Medicine, Tzu Chi University, Hualien 970, Taiwan; 5 Department of Orthopedics, Taipei Tzu Chi Hospital, Buddhist Tzu Chi Medical Foundation, New Taipei City 231, Taiwan

**Keywords:** scoliosis, knee osteoarthritis, total knee replacement

## Abstract

**Background/Objectives**: Scoliosis has been linked to pelvic position and tilt angle, but few studies have explored its relationship with knee pathology, which can be a significant burden for the elderly. Our aim is to investigate the relationship between scoliosis and knee osteoarthritis (OA). **Methods**: This population-based case-control study using data from the National Health Insurance Database of Taiwan included patients diagnosed with OA who underwent total knee replacement (TKR) for the first time between 2014 and 2019. Propensity score matching was employed to select controls who did not undergo TKR. Scoliotic cases were identified if they had been diagnosed before knee OA and TKR. The study samples included 10,021 patients with OA/TKR and 30,063 patients without OA/TKR. We then utilized logistic regression analysis to calculate the odds ratio (OR) and 95% confidence interval (CI) for the diagnosis of scoliosis prior to the index date. **Results**: The findings indicate that patients with pre-existing scoliosis have a 1.627 times greater likelihood of undergoing TKR, with significantly higher odds ratios observed across both female (OR = 1.583) and male (OR = 1.909) and younger (OR = 2.102) and older (OR = 1.575) patients. However, a notable limitation of this study is the absence of data on scoliosis curve side, which prevented us from analyzing the relationship between curve direction and knee arthritis laterality. Furthermore, while we included diagnostic codes indicating left or right TKR, the lack of precise measurements for variables such as lower limb length discrepancies may introduce residual confounding. **Conclusions**: Our research suggests a significant association between scoliosis and the development of knee OA.

## 1. Introduction

Scoliosis is an atypical sideways curvature of the spine [1]. It generally occurs in children and adolescents, but the exact factors for the beginning of the curve remain unknown [2]. Adults may develop scoliosis as well, and common types include de novo adult degenerative scoliosis (ADS), with progression of congenital, early onset, or adolescent idiopathic scoliosis [1]. Unlike progressive idiopathic scoliosis, ADS is a chronic condition resulting from musculoskeletal degeneration and commonly localized to the lumbar spine [1].

Osteoarthritis (OA) of knee is one of the main causes for impairments among adults older than 65 years [3]. Although OA is generally associated with aging, research indicates that younger individuals can also experience osteoarthritic changes. Currently, over 527 million people worldwide are estimated to be affected by OA, and as the population continues to age, this number is expected to rise significantly [4]. OA primarily affects the articular cartilage (AC), a specialized tissue that facilitates smooth, nearly frictionless joint movement. As OA progresses, this cartilage deteriorates, impacting not only the cartilage itself but also additional joint structures such as the bone, synovium, and surrounding ligaments. Structural changes are particularly evident in weight-bearing zones of the cartilage, where OA can lead to microcracks that gradually extend into deeper layers. In particular, OA of the knee is common and often leads to severe pain, stiffness, and functional limitations [4,5]. Studies have identified several risk factors for knee OA, including age, female gender, obesity, knee injury, sports-related activities, occupational stress such as frequent bending and lifting, and lower limb misalignment such as varus or valgus knee alignment. Obesity, in particular, not only heightens the risk of OA but also exacerbates symptoms [5,6]. The socioeconomic impact of OA is substantial, with related healthcare and productivity costs reaching up to 2.5% of a country’s GDP [4]. As the disease progresses, pain, reduced joint motion, and muscle weakness can hinder physical activity and increase the risk of fatigue, depression, and disability [5,6]. Unendurable pain of knees is the main reason when people search for knee surgeries [3]. Total knee replacement (TKR) is one of the most common knee surgeries [7], and over 97% of knee arthroplasties are performed due to knee OA [3]. Many conservative treatments are available for early knee OA; however, when facing end-stage knee OA, total knee replacement is the preferred treatment [8]. Therefore, an important criterion for assessing the severity of knee OA is determining whether TKR surgery is necessary.

Previous studies have explored the relationship between scoliosis and pelvic position, tilt angle, and even hip joint dislocation [9,10]. Further studies also claim that position and posture of pelvis are closely related to the load and control of the knee joint [11,12,13]. Scoliosis may cause changes in coronal spinopelvic alignment, leading to changes in pressure and internal moments of force on the knee joints [14]. This can ultimately elevate the risk of knee joint injuries and, furthermore, exacerbate the severity of arthritis. However, few studies have explored the relationships between scoliosis and its increased risk of severe knee OA that requires knee joint-related surgeries. The objective of this study is to investigate the association between scoliosis and the progression of knee osteoarthritis, specifically examining whether scoliosis increases the likelihood of severe knee OA that necessitates TKR. Scoliosis, by altering the coronal and sagittal alignment of the spine, may shift the body’s weight distribution and place additional stress on the lower extremities, particularly the knee joints. This misalignment can lead to abnormal force patterns and increased load on the knee’s articular cartilage, potentially accelerating OA progression. Furthermore, changes in pelvic tilt and posture associated with scoliosis may affect knee stability and exacerbate structural deterioration, compounding OA-related joint damage. Investigating this relationship is important, as understanding how scoliosis contributes to knee OA could provide critical insights into managing and potentially mitigating OA severity in patients with spinal curvature abnormalities. Clinically, identifying scoliosis as a contributing factor could help healthcare providers tailor early intervention strategies, reducing the need for invasive procedures like TKR and, ultimately, improving patient outcomes and quality of life. With TKR surgeries accounting for a significant portion of healthcare costs and having long-term implications for patients, the findings from this research could support cost-effective, preventative approaches that address the underlying biomechanical connections between scoliosis and OA.

## 2. Materials and Methods

### 2.1. Database

This case-control study used data from Taiwan’s Longitudinal Health Insurance Database 2005 (LHID 2005), which comprises medical claims and registration files for a randomly selected sample of 2,000,000 enrollees under the Taiwan National Health Insurance (NHI) program. The Ministry of Health and Welfare of Taiwan randomly selected these 2,000,000 enrollees from all beneficiaries listed in the 2005 Registry of Beneficiaries, including anyone who was a beneficiary of the NHI program during any period in 2005. Therefore, the LHID 2005 enables researchers to track medical services provided to the selected enrollees longitudinally since 2000.

The study received approval from the Institutional Review Board of Taipei Tzu Chi Medical Center (Protocol No.: 12-W-045) and adhered to the principles of the Declaration of Helsinki. Informed consent was not required as our research used de-identified administrative data.

### 2.2. Identification of Patients

To select the patients for the case group, we retrieved data on 10,021 patients who were diagnosed with knee osteoarthritis (OA) (ICD-9: 715.16, 715.26, 715.36, 715.96 or ICD-10: M17.1, M17.5, M17.9) and underwent total knee replacement (TKR) (ICD-9: 81.54 or ICD-10: OSRC072, OSRC0J9, OSRC0JA, OSRC0JZ, OSRC0KZ, OSRD07Z, OSRD0J9, OSRD0JA, OSRD0JZ, OSRD0KZ, OSRT07Z, OSRT0JZ, OSRT0KZ, OSRU07Z, OSRU0JZ, OSRU0KZ, OSRV07Z, OSRV0JZ, OSRV0KZ, OSRW07Z, OSRW0JZ, OSRW0KZ) for the first time between 1 January 2014 and 31 December 2019. To ensure the accuracy of knee OA diagnosis, we defined patients who underwent TKR surgery as having severe knee OA, since we did not have information about stages of knee OA in this dataset. We also excluded cases under 20 years old. Given that administrative datasets are often criticized for their poor diagnostic validity, we further revised our screening criteria to include patients undergoing TKR and excluded those who had TKR without a diagnosis of knee OA. We assigned the date of receiving their first TKR surgery as the index date for those in the case group.

**Figure 1:**
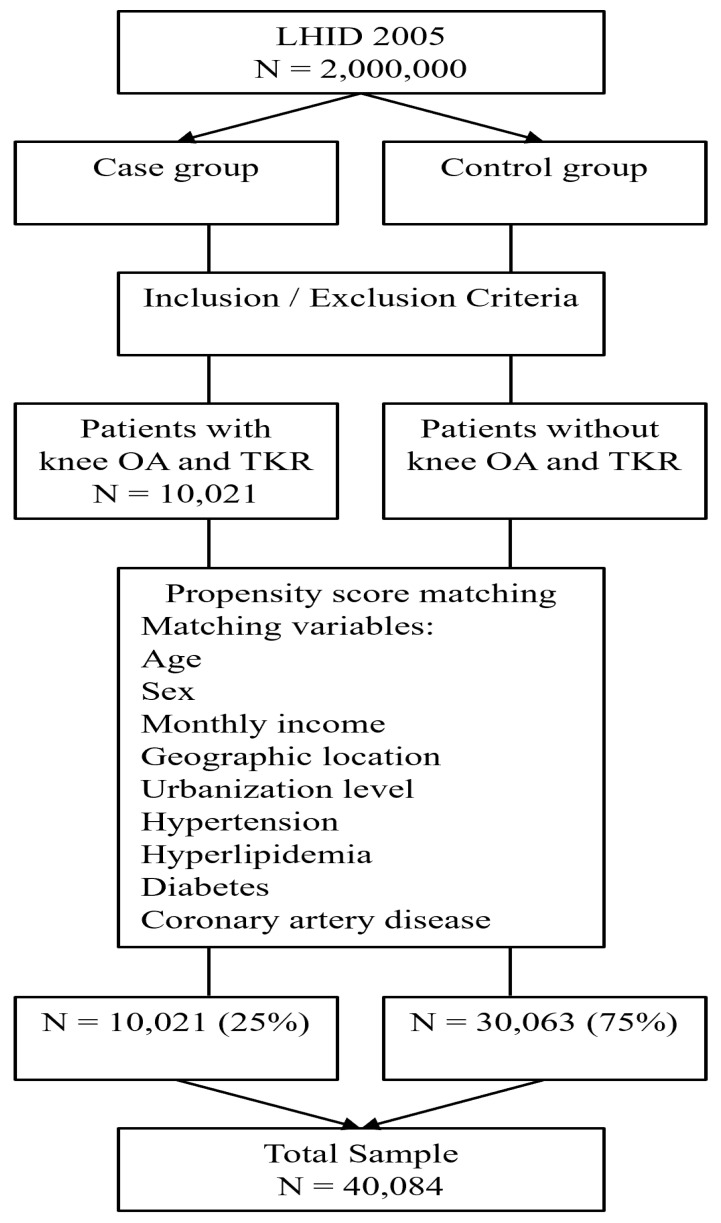
Flow diagram of the study sample.

The controls for the comparison group were also identified from the remaining LHID 2005 enrollees. We excluded all participants with a history of knee OA who had undergone TKR from 2000 to 2019, and then used the propensity score matching method, which was calculated by using the logistic regression model, with adjustment for age, sex, monthly income categories, geographic location, urbanization level of the patient’s residence, and common medical conditions including hypertension, hyperlipidemia, diabetes, and coronary artery disease. Income, geographic location, and urbanization level were included based on previous research suggesting that socioeconomic factors may influence both healthcare access and the prevalence of musculoskeletal conditions, including knee OA. These factors were included to control for potential disparities in healthcare access and regional variations in OA management. Finally, with a 1:3 case-to-control ratio and nearest neighbor random matching algorithm (predefined caliper range of ±0.01), a total of 30,063 controls were randomly selected (Figure 1). The index date for the control group corresponded to that of their respective matched case, since they did not receive knee surgery.

### 2.3. Measures of Outcomes

All scoliotic cases were identified using ICD-9 code 737.3 and ICD-10 code M41 in their diagnosis, and we focused only on cases that had been diagnosed at least twice by either orthopedists or rehabilitation physicians, as this was an experiment where we first looked for the later stages and then went back to investigate the earlier stages. Scoliotic cases were identified if they had been diagnosed before the index date. This approach was taken to enhance the reliability of having a scoliotic condition as obtained from the administrative database.

### 2.4. Statistical Analysis

Statistical analyses were performed using the SAS system (SAS System for Windows, Version 8.2, SAS Institute Inc., Cary, NC, USA). To compare the distribution of demographic variables and medical comorbidities between cases and controls, chi-square tests were conducted. This method is commonly employed to assess the relationship between categorical variables and determine whether significant differences exist among groups [15]. Logistic regression analysis was utilized to calculate the odds ratio (OR) and 95% confidence interval (CI) for the diagnosis of scoliosis prior to the index date between cases and controls. This regression technique is appropriate for examining the influence of multiple independent variables on a binary outcome, allowing for the adjustment of potential confounders, including demographic variables and medical comorbidities [16]. Statistical significance was evaluated using a conventional two-tailed *p*-value threshold of ≤0.05. This criterion is standard in clinical research for determining whether observed associations are likely due to chance [17]. The combination of chi-square tests and logistic regression provides a robust framework for understanding the relationship between scoliosis and knee osteoarthritis, contributing valuable insights with implications for patient management and treatment strategies.

## 3. Results

The demographic variables and medical comorbidities of the patients are summarized in Table 1. After applying propensity score matching, the demographic characteristics of the matched groups were similar. The mean age of patients with knee OA who underwent TKR (case group) was 72.99 ± 8.52 years, compared to 73.12 ± 8.67 years in the control group (*p* = 0.194). The sex distribution was balanced, with no statistically significant difference between cases and controls (*p* = 1.0000). Income levels (*p* = 0.5919) and urbanization status (*p* = 0.8014) were also comparable across groups. However, a statistically significant difference was observed relating to geographic location (*p* < 0.0001), with the largest disparity noted in the eastern region, potentially attributed to its smaller population size or differing lifestyles. Regarding medical comorbidities, all factors were statistically similar for both groups, including hypertension (*p* = 1.0000), hyperlipidemia (*p* = 1.0000), diabetes (*p* = 1.0000), and coronary artery disease (*p* = 1.0000).

**Figure 2:**
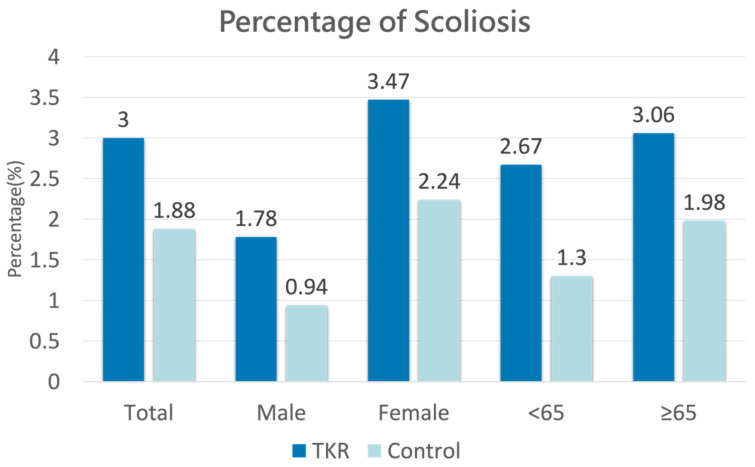
Percentage of pre-existing scoliosis cases among case and control groups.

Figure 2 illustrates the prevalence of pre-existing scoliosis among both cases and controls. Among the 20,021 patients with OA/TKR, 3.0% (N = 301) had a prior diagnosis of scoliosis. In comparison, out of 30,063 patients in the control group, only 1.88% (N = 565) had been diagnosed with scoliosis before the index date. For the male cohort, 1.78% (N = 49) of the 2758 OA/TKR patients had a prior scoliosis diagnosis, while only 0.94% (N = 78) of the 8274 patients in the control group had been diagnosed. In the female cohort, 3.47% (N = 252) of the 7263 OA/TKR patients had scoliosis prior to the index date, compared to 2.24% (N = 487) of the 21,789 control patients. In the group aged under 65, 2.67% (N = 41) of the 1538 OA/TKR patients were diagnosed with scoliosis before the index date, whereas only 1.30% (N = 60) of the 4614 patients in the control group had a prior diagnosis. For those aged over 65, 3.06% (N = 260) of the 8483 OA/TKR patients had scoliosis, compared to 1.98% (N = 505) of the 25,449 control patients.

The odds ratios (ORs) and their corresponding 95% confidence intervals (CIs) for previous scoliosis diagnosis between cases and controls are detailed in Table 2. Conditional logistic regression, adjusting for sex, age, and index year, yielded a crude OR for pre-existing scoliosis in cases of 1.617 (95% CI: 1.403–1.863) compared to controls. After adjusting for geographic region, monthly income, urbanization level, and comorbidities (including hypertension, hyperlipidemia, diabetes, and coronary artery disease), the adjusted OR for pre-existing scoliosis in the case group was 1.627 (95% CI: 1.411–1.876) relative to the control group.

Table 3 further categorizes the ORs for previous scoliosis diagnoses by gender. Male cases exhibited a notably higher adjusted OR for scoliosis diagnosis prior to the index date than controls (OR 1.909; 95% CI: 1.331–2.737). Similarly, female cases also showed a significant difference in the adjusted OR for pre-existing scoliosis compared to the control group (OR 1.583; 95% CI: 1.356–1.848). These findings suggest that both male and female patients with knee OA who undergo TKR may have a heightened risk for scoliosis, which could influence surgical outcomes and rehabilitation approaches.

When cases were stratified by age, elderly patients (≥65 years) had a significantly adjusted OR for pre-existing scoliosis of 1.575 (95% CI: 1.352–1.834) in the case group compared to controls. Notably, younger patients (<65 years) exhibited an even higher adjusted OR of 2.102 (95% CI: 1.406–3.143) for pre-existing scoliosis. This suggests that both older and younger patients undergoing TKR may require targeted screening and management strategies to address scoliosis, which could impact their surgical recovery and long-term outcomes (Table 4).

## 4. Discussion

This study highlights that patients with pre-existing scoliosis have 1.627 times the odds of undergoing subsequent TKR surgery. In other words, scoliosis is highly associated with an increased risk of developing severe knee OA. The results are significant across all subgroups, including age and gender. The adjusted OR is 1.901 for the male group, 1.583 for the female group, 1.575 for those aged ≥ 65, and 2.102 for those aged < 65. To date, most previous studies investigating the relationship between scoliosis and orthopedic diseases have focused only on the pelvic region [9,10,18]. Other studies primarily focused on hip OA and knee OA leading to truncal changes [19,20]. Conversely, few studies have explored the impact of scoliosis on knee joints. Márkus et al. [14] showed that patients with decompensated adolescent idiopathic scoliosis have alterations in the biomechanical parameters of lower limbs, with a reduced collodiaphyseal angle and increased varus deformity at the knee joints. Our aim is to fulfill this research gap by using a nationwide database.

The main features of scoliosis include formation of curves on the coronal alignment spine, and some extent of sagittal deformation and rotational anomaly, which are often intercorrelated [21,22]. The close relationship between low back pain and knee osteoarthritis has been widely accepted [23]. Additionally, many previous studies have also suggested the association of knee OA and sagittal malalignment of the spine [24,25]. However, to the best of our knowledge, only a few studies have confirmed the relationship between coronal malalignment of the spine and knee OA. Du et al. [26] investigated the interaction between the coronal plane of the spine and knee OA and found a significant correlation between the two.

The prominence of coronal malalignment of the spine in scoliotic patients can cause imbalance in the coronal plane and subsequently result in pelvis compensation, which affects the muscles of the lower extremities and the progression of knee OA [26,27,28]. On the other hand, with the progression of spinal deformity, pelvic tilt also increases, which in turn exacerbates vertebral stress and worsens spinal malalignment and knee OA, leading to pain and knee joint dysfunction [28,29]. Du’s [26] study found that the deviation of vertebral distance from the midline, and changes in the center of gravity due to coronal malalignment, affects WOMAC pain score and function score, which corresponds to the results of our study that scoliosis may be an independent cause of knee OA.

Scoliosis should be viewed as a three-dimensional deformity; in addition to coronal malalignment, these patients also demonstrate sagittal anomaly of the spine. Poor spinopelvic alignment has been shown to correlate with the severity of knee OA [24,25]. Specifically, lumbo-pelvic malalignment with poor lumbar lordosis and pelvic retroversion are also strongly related with knee OA [24,30]. The mechanism may include compensation of poor lumbar lordosis and subsequent knee flexion and thigh muscle fatigue, which further induces patellofemoral joint pain [31]. From another perspective mentioned by Sato et al. [32], it was demonstrated that surgery for spinal correction not only improved spine and lower extremity alignments, but also enhanced patients’ quality of life, with particularly significant effects observed in patients with mild knee OA.

Studies have also shown that patients with concomitant knee OA and lumbar spinal stenosis have poorer outcomes after knee replacements compared to those without spinal disorders [33]. Ozaki et al. [34] found that the surgical outcome of lumbar stenosis with knee OA was poorer than those without knee OA. This evidence shows a strong correlation between these two disorders. The results of our study offer insight regarding the increased risk of surgical intervention for knee OA in patients with scoliosis, and highlight the importance of being aware of possible degenerative knee arthritis when patients are diagnosed with scoliosis. Comprehensive assessment should be applied to patients with scoliosis, including factors such as higher body weight, abnormal knee alignment, poor joint stability, and inadequate muscle strength, in order for their doctors to take early intervention action to prevent further progression of knee OA.

While our study demonstrates that scoliosis is associated with a higher risk of severe knee OA, the possibility of reverse causality should be considered. Currently, there is no definitive evidence that early knee OA symptoms directly cause scoliosis. However, there may be some indirect connections between the two conditions. For example, postural changes due to early OA can lead to pain and joint stiffness, which might alter a person’s posture and gait. These postural changes could place asymmetric stress on the spine, potentially affecting its alignment. Another consideration is functional changes. Early knee OA might cause functional changes, such as altered gait and reduced mobility, which could impact the spine and potentially lead to scoliosis. Further studies are required to disentangle these relationships and better understand whether knee OA could influence spinal alignment and contribute to the development or worsening of scoliosis.

This study has strength in several key aspects. First, the adoption of a longitudinal, population-based database effectively minimizes selection bias and mitigates recall bias, which are frequently encountered in case-control studies. Second, the fact that over 98% of Taiwan’s population is of Chinese Han ethnicity means that the study benefits from a relatively uniform population, reducing the risk of racial confounding factors. Third, the study uses a population-based dataset with a large sample size, which provides a significant statistical advantage for detecting real differences in the prevalence of pre-existing scoliosis between patients who underwent TKR and those who did not. This large sample size gives us strong confidence in exploring the association between scoliosis and knee OA during the study period. Fourth, the diagnosis of scoliosis has high validity, as we only considered cases that had been diagnosed at least twice, by either certified orthopedists or rehabilitation physicians.

However, there are still several limitations in this study. First, we could only use ICD codes for selected cases with scoliosis and knee OA, and imaging data such as Cobb angles of scoliosis or grading for knee OA were not able to be obtained. These are definitely crucial details for evaluating the severity of both conditions. Nevertheless, since this information was not available in the database, we defined the patients undergoing TKR surgery as having severe knee OA. Moreover, we acknowledge that any missing data, particularly in administrative databases, could introduce bias. Second, the database also provides no lifestyle information or laboratory records, including personal lifestyle, body weight, BMI, inflammatory biomarkers, family history, or genetic factors. These omissions may impact the understanding of the relationship between these factors and knee OA. Specifically, personal lifestyle factors such as body weight and physical activity are crucial, as obesity and lack of exercise can significantly contribute to the risk of knee OA. Inflammatory biomarkers are important because chronic inflammation is known to play a role in the progression of knee OA, with elevated levels of markers like C-reactive protein and cytokines (e.g., IL-6) often being observed in osteoarthritic patients. Additionally, family history and genetic factors may be relevant, since positive family history of knee OA and specific genetic variations can increase susceptibility to the disease. Third, our database covers records from 2000 to 2019, and since scoliosis-induced coronal malalignment is a chronic process, we are not able to access information on scoliosis diagnosed prior to 2000. Due to the above-mentioned limitations, we were not able to obtain the exact duration between initial diagnosis of scoliosis and the date of TKR surgery, which would have provided some insights into the progression of disorder. Fourth, a potential limitation of this study is the exclusion of detailed lower limb length discrepancies (LLD) data, which could impact our findings. LLD is known to affect biomechanics, potentially contributing to conditions such as scoliosis and knee OA, which are both relevant to our analysis. In cases where patients have both scoliosis and knee OA, LLD could serve as a confounding factor, influencing the progression or severity of each condition independently. Thus, failure to account for LLD may introduce bias into our results. While our database includes diagnostic codes for LLD, it lacks precise measurements, such as the centimeter difference between limbs. Without these specifics, it is challenging to assess the extent of LLD or accurately determine its impact. Including LLD in our analysis without this granularity could lead to misclassification bias or inaccurate effect estimates. Moreover, LLD coding is uncommon under Taiwan’s NHI programs, and only a small number of OA/TKR patients are coded with LLD. Incorporating LLD into the analysis would require further stratification, which, given the limited coding, would result in a low sample size and potentially biased or unanalyzable results. However, we acknowledge that not accounting for LLD may leave some residual confounding. Future research that includes precise LLD measurements could more thoroughly examine its role in scoliosis and knee OA, leading to a clearer understanding of its impact. Fifth, another limitation of this study is the lack of data on the side of the scoliosis curve, which prevented us from analyzing the relationship between the curve side and the side of knee arthritis. Although the database includes information on whether patients underwent left or right TKR, it does not specify the curve direction for scoliosis. This limitation restricts our ability to examine potential associations between the lateralization of scoliosis and knee arthritis, which could provide insights into biomechanical or compensatory patterns affecting joint health. Future studies with access to detailed scoliosis curve data could enhance understanding of the interaction between scoliosis and knee arthritis side, potentially revealing important lateralized effects. Lastly, our study lacks in-depth between-group analysis by different age and gender subgroups. Our primary focus was to explore the overall association between scoliosis and severe knee osteoarthritis, rather than examining how different demographic subgroups might uniquely impact this association. Future studies with larger, more stratified sample sizes and more detailed data could provide valuable insights by investigating between-group differences to further enhance the clinical relevance of these findings.

When it comes to early intervention to prevent the progression of knee OA, Sánchez Romero et al. [35] highlight that long-term outcomes for knee OA in patients with prior ACL injuries are influenced by lifestyle modifications and bone contusions, underlining the importance of targeted prevention and rehabilitation strategies to mitigate OA progression. Considering the potential impact of gender and movement pattern differences on knee health, Prados-Barbero et al. [36] highlight the importance of movement patterns, hip mobility, and neuromuscular control in maintaining knee stability, particularly in preventing excessive knee valgus, a movement that places added stress on the knee joint. Their study underscores the value of developing training and rehabilitation programs tailored by gender to improve neuromuscular control and reduce knee stress. These findings are relevant to scoliosis patients, who may exhibit abnormal movement patterns that affect knee health. Rehabilitation programs for scoliosis patients could therefore focus on enhancing lower limb stability, increasing hip range of motion, and strengthening core muscles to support the knee’s mechanical demands, potentially reducing the progression risk of knee osteoarthritis.

## 5. Conclusions

This study underscores the significant association between scoliosis and severe knee OA requiring TKR. Our findings indicate that patients with pre-existing scoliosis have a 1.627 times greater likelihood of undergoing TKR, with significantly higher odds ratios observed across both female and male and younger and older patients. This association suggests that scoliosis, through its impact on spinal alignment and biomechanics, may accelerate knee joint degeneration, contributing to OA progression. As scoliosis alters the body’s coronal and sagittal balance, it increases stress on the lower extremities, leading to abnormal load distribution on the knee’s articular cartilage and potentially exacerbating OA.

The study’s strength lies in its use of a population-based dataset with a large sample size, enhancing the reliability of the findings. However, limitations such as the lack of imaging data and detailed lifestyle or body composition information may affect the accuracy of certain associations. Despite these limitations, our research emphasizes the importance of comprehensive screening for knee OA in patients with scoliosis, particularly those at higher risk due to factors such as obesity, abnormal knee alignment, joint instability, or poor muscle strength. Clinicians should consider targeted intervention strategies, including physical therapy focused on improving lower limb stability, hip mobility, and core strength, to mitigate OA progression in this population. Early intervention is recommended to prevent further progression of knee OA.

Despite advances made in understanding this topic, the specific pathomechanisms behind this relationship remain unclear. Further research is needed to corroborate this association and to understand the underlying mechanisms. Future studies incorporating detailed imaging data, lifestyle factors, and assessments of lower limb length discrepancies could provide more nuanced insights into the scoliosis–knee OA relationship, leading to refined intervention strategies. This study highlights the need for early intervention and rehabilitation strategies tailored to the unique biomechanical challenges faced by scoliosis patients to ultimately reduce the risk and severity of knee OA and enhance patient outcomes and quality of life.

## Figures and Tables

**Table 1:** Demographic data.

Variable	Patients with Knee OA and Underwent TKR (n = 10,021)		Controls (n = 30,063)		*p* Value
	n	%	n	%	
Age, mean (SD)	72.99(8.52)		73.12(8.67)		0.1940
Sex					1.0000
Males	2758	27.52	8274	27.52	
Females	7263	72.48	21,789	72.48	
Income ^†^					0.5919
NT$1–15,841	2051	20.47	6031	20.06	
NT$15,841–25,000	5148	51.37	15,444	51.37	
NT$ > 25,001	2822	28.16	8588	28.57	
Area					<0.0001
Northern	4038	40.30	12,087	40.21	
Central	2524	25.19	7724	25.69	
Southern	3115	31.08	9601	31.94	
Eastern	344	3.43	651	2.17	
Urbanization ^‡^					0.8014
Urb-1	2140	21.36	6379	21.22	
Urb-2	2417	24.12	7414	24.66	
Urb-3	1680	16.76	5078	16.89	
Urb-4	1826	18.22	5420	18.03	
Urb-5	1958	19.54	5772	19.20	
Hypertension	7953	79.36	23,859	79.36	1.0000
Hyperlipidemia	6024	60.11	18,072	60.11	1.0000
Diabetes	4347	43.38	13,041	43.38	1.0000
Coronary heart disease	3364	33.57	10,092	33.57	1.0000

^†^ In 2024, the average exchange rate was US$1 ≈ NT$32. ^‡^ Urb-1 = most urbanized, Urb-5 = least urbanized. SD: standard deviation. NT$ = Taiwanese new dollar.

**Table 2:** Odds ratio between case group and control group.

	Total (N = 40,084)		Patients with Knee OA and Underwent TKR (n = 10,021)		Controls (n = 30,063)	
Presence of Scoliosis	n	%	n	%	n	%
Yes	866	2.16	301	3.00	565	1.88
No	39,218	97.84	9720	97.00	29,498	98.12
Crude OR ^†^ (95% CI)	-		1.617 *** (1.403–1.863)		1.00	
Adjusted OR ^‡^ (95% CI)	-		1.627 *** (1.411–1.876)		1.00	

^†^ Crude OR was calculated using conditional logistic regression analysis conditioned on sex, age, and year of index date. ^‡^ Adjustment for patient’s sex, age, and year of index date, geographic region, urbanization level and medical comorbidities. *** *p* < 0.001. CI = confidence interval; OR = odds ratio.

**Table 3:** Stratification according to patient gender.

	Male		Female	
	Patients with Knee OA and Underwent TKR (n = 2758)	Controls (n = 8274)	Patients with Knee OA and Underwent TKR (n = 7263)	Controls (n = 21,789)
Presence of Scoliosis	n (%)		n (%)	
Yes	49 (1.78%)	78 (0.94%)	252 (3.47%)	487 (2.24%)
Crude OR ^†^ (95% CI)	1.901 *** (1.326–2.724)	1.00	1.572 *** (1.347–1.835)	1.00
Adjusted OR ^‡^ (95% CI)	1.909 *** (1.331–2.737)	1.00	1.583 *** (1.356–1.848)	1.00

^†^ Crude OR was calculated using conditional logistic regression analysis conditioned on sex, age, and year of index date. ^‡^ Adjustment for patient’s sex, age, and year of index date, geographic region, urbanization level and medical comorbidities. *** *p* < 0.001. CI = confidence interval; OR = odds ratio.

**Table 4:** Stratification according to patient age.

	<65		≥65	
	Patients with Knee OA and Underwent TKR (n = 1538)	Controls (n = 4614)	Patients with Knee OA and Underwent TKR (n = 8483)	Controls (n = 25,449)
Presence of Scoliosis	n (%)		n (%)	
Yes	41 (2.67%)	60 (1.30%)	260 (3.06%)	505 (1.98%)
Crude OR ^†^ (95% CI)	2.079 *** (1.392–3.106)	1.00	1.562 *** (1.342–1.818)	1.00
Adjusted OR ^‡^ (95% CI)	2.102 *** (1.406–3.143)	1.00	1.575 *** (1.352–1.834)	1.00

^†^ Crude OR was calculated using conditional logistic regression analysis conditioned on sex, age, and year of index date. ^‡^ Adjustment for patient’s sex, age, and year of index date, geographic region, urbanization level and medical comorbidities. *** *p* < 0.001. CI = confidence interval; OR = odds ratio.

## Data Availability

All register information was provided by the Taiwan National Health Research Institute. Information about how to apply for data from Taiwan health care registers is available at http://www.nhri.org.tw (accessed on 6 October 2024).
